# Teucer Simmons Fires from behind the Shield of Ajax

**Published:** 1907-11

**Authors:** 


TEUCER SIMMONS FIRES FROM BEHIND THE SHIELD OF AJAX.—Screened and protected by the Board of Trustees and the self-constituted Council of Pharmacy,—the Power behind the Octopus,—the autocrat who pulls the string is case-mated, bomproof, not get-at-able. As in the days of the Inquisition,—anyone falling under the displeasure of the Power may be haled up, tried with closed doors, doomed and executed "without the benefit of clergy," without the privilege of reply or defense. Dr. Abbott, of the Alkaloidal Company, has been so treated—all but the execution, and has been denied the privilege of defense. It is a shame and an outrage, and had not the entire independent medical press submitted to an outrageous indignity at the hands of this Octopus in the matter of having the said journals denounced as "unclean" and excluded from the exhibition hall;—and had not the recalcitrant but now recidivous Coe, who was looked to to second the "Red Back's" protest and lead a revolt of the medical editors, joined in the "unanimous approval" of the Octopus management, I would be tempted, almost, to hope and say that the medical profession would not stand for such treatment, but would demand a square deal. They will stand for anything at the hands of the monster Octopus!

But Dr. Edwards, of the *Va. Med. Semi-Monthly,* tells the tale better than I can do, and I reproduce his editorial in full, and add my hearty endorsement of it. Here it is:

COUNCIL ON PHARMACY AND CHEMISTRY VERSUS ABBOTT ALKALOIDAL COMPANY.

"Fair play" is a demand of the times—in fact, it is inherent in the human breast. Let the "umpire" of a baseball team, or the "referee" of a football game make a biased decision, and he is jeered by the crowd of spectators. If a college professor is grossly unjust in his treatment of a student, the student body rises up in complaint. Not even the present, all-powerful American Medical Association, or those in charge of its affairs, can afford to be unjustly dictatorial in its ways without the risk of discord among its members, or even of disintegration of the Association itself.

It seems that some months ago the Council on Pharmacy and Chemistry criticised some of the preparations of the Abbott Alkaloidal Company. Dr. W. C. Abbott, who is a member of the Association, as also of the Alkaloidal Company, prepared and sent the *Journal A. M. A.* a reply to the criticisms, which that journal declined to publish—even as a paid advertisement. Such decision of those in charge of the *Journal* has forced Dr. Abbott to the necessity of issuing a circular in self-explanation at the expense of his firm, and which, of course, places him at great disadvantage.

The controversy, it would appear, grew out of publications by the Committee on Pharmacy and Chemistry concerning "Calcidin," the "hyoscine-morphine-cactin" compound and perhaps other preparations of the Abbott Alkaloidal Company. With reference to "Calcidin," which is an iodine compound, it is recognized that a chemical change goes on in it all the time, but the company claims that its therapeutic efficiency is not thereby materially affected, and refers to the continued professional demand for it, in support of their contention. It appears, further, that Dr. Abbott made a monetary offer for any one to help him to hold "Calcidin" to its original state as sent out from the laboratory. Dr. Abbott still further offered to show any member of the Council or any accredited representative thereof just exactly how the preparation is made.

It is needless to go over the controversy between the Council and the Abbott Alkaloidal Company, about the "hyoscine-cactin-morphine compound tablets," for the history is much the same as that above about "Calcidin."

But the point of the whole matter is that if Dr. Abbott is a member of the Association, if he has added anything to pharmacy or to therapeutics by the introduction and popularization of products accepted and fully tested by the profession—and favorably tested also; if the preparations are not of secret composition, and have only the protection of proprietary rights; if he has offered to demonstrate before the Council or any of its authorized representatives the details of manufacture; if such preparations have, in his opinion, been officially wrongly criticised in the *Journal;* and if the *Journal* declined to publish any respectfully worded reply to such criticisms so vitally affecting his interests, then "it is time to kick." Who knows who will be the next victim? We insist upon "fair play," and we are sure that many other members of the American Medical Association throughout the United States will agree with us.—*Va. Med. Semi-Monthly, Oct.,* 1907.

